# Creating Surface Properties Using a Palette of Hydrophobins

**DOI:** 10.3390/ma3094607

**Published:** 2010-09-06

**Authors:** Filippo Zampieri, Han A. B. Wösten, Karin Scholtmeijer

**Affiliations:** 1 Microbiology, Institute of Biomembranes, Utrecht University, Padualaan 8, 3584 CH Utrecht, The Netherlands; E-Mails: F.Zampieri@uu.nl (F.Z.); H.A.B.Wosten@uu.nl (H.A.B.W.); 2 BiOMaDe Technology Foundation, Nijenborgh 4, 9747 AG Groningen, The Netherlands; 3 Department of Microbiology, Groningen Biomolecular Sciences and Biotechnology Institute (GBB), University of Groningen, PO Box 14, 9750 AA Haren, The Netherlands

**Keywords:** hydrophobin, self-assembly, wettability, coating of surfaces, immobilization

## Abstract

Small secreted proteins called hydrophobins play diverse roles in the life cycle of filamentous fungi. For example, the hydrophobin SC3 of *Schizophyllum commune* is involved in aerial hyphae formation, cell-wall assembly and attachment to hydrophobic surfaces. Hydrophobins are capable of self-assembly at a hydrophilic-hydrophobic interface, resulting in the formation of an amphipathic film. This amphipathic film can make hydrophobic surfaces of a liquid or a solid material wettable, while a hydrophilic surface can be turned into a hydrophobic one. These properties, among others, make hydrophobins of interest for medical and technical applications. For instance, hydrophobins can be used to purify proteins from complex mixtures; to reduce the friction of materials; to increase the biocompatibility of medical implants; to increase the solubility of water insoluble drugs; and to immobilize enzymes, for example, biosensor surfaces.

## 1. Introduction

Metals, ceramics, carbon and polymers are attractive materials for use in applications such as biosensors, microarrays, medical implants and cell culturing. Surface modification is often the key to successful use of these compounds [1,2,3]. Surface modification is a process that changes the material surface composition, structure and morphology. The intrinsic mechanical properties are left intact while the biofunctionality and/or the biocompatibility of the material increases [4]. This results in a change in the physical micro-architecture of the surface, a change in biochemical properties, and/or a change in the visco-elastic properties [1,2,5].

Conventional surface modification techniques make use of dry processes (e.g., using beams of ions or electrons [3,6,7]) or wet processes (using aqueous solutions) [1,4]. In both cases, the surface modification involves either physical (van der Waals’ type) or chemical adsorption of compounds [4,7]. Examples of chemical adsorption are the use of self-assembled monolayers (SAMs) such as aminosilane and epoxysilane or the use of nitrocellulose to modify the surface of silica glass in DNA-microarrays [8]. On the other hand, protein coatings are exploited for their affinity for specific ligands as in protein chips [8]. Alternatively, three dimensional hydrogels can be used to physically entrap molecules in their matrix (e.g., in drug delivery systems and biosensors) [5]. These non-covalent or a-specific interactions (hydrogen bonds, van der Waals forces, ionic bonds and hydrophobic interactions) are generally applicable [8,9]. Adsorption via covalent bonds (also called “true chemical adsorption”) can be used for instance to control the structure, stability and thickness of the modified surface [8,9].

Surface modification via non-covalent adsorption of proteins often involves loss of tertiary structure and therefore loss of biological activity [10]. To overcome this, proteins are usually covalently immobilized through introduced reactive groups (e.g., hydroxyl, carboxyl and amino groups). Examples are the use of covalently linked adhesive proteins derived from the extracellular matrix (ECM) of human or animal tissue (e.g., fibronectin, laminin, vitronectin, collagen) that promote cell adhesion, or the use of immobilized growth factors that modulate cell proliferation and differentiation [5,11]. Hydrophobins offer an alternative for these methods. These surface-active fungal proteins adsorb non-covalently to the material. Yet, they can form a highly stable coating which can be used to promote biocompatibility, to improve stability and particle size of suspensions and emulsions, or to preserve the activity of proteins at a surface of a liquid or a solid material [12,13,14,15]. In this review the function, structure and self-assembly of hydrophobins is discussed as well as their potential use in technical and medical applications.

## 2. Biological Functions of Hydrophobins

Hydrophobins play a key role in growth and morphogenesis in the majority of the filamentous fungi [12,15,16,17]. Their functions are mainly based on their capability to self-assemble into a highly surface active film at a hydrophilic-hydrophobic interface [18,19,20]. Although hydrophobins show differences in their primary sequence, they share eight conserved cysteine residues that form four disulphide bridges [15,16]. Based on the spacing of the cysteine residues and their biophysical properties, hydrophobins can be divided in two classes [21]. So far, class II hydrophobins have been observed only in Ascomycetes, whereas class I hydrophobins are produced both in Ascomycetes and Basidiomycetes [15,16].

Filamentous fungi grow into the air to form sexual and a-sexual reproductive structures, the most conspicuous structures being the mushrooms. The water surface tension makes the interface between the moist substrate and the air a barrier for fungi to grow into the air. Fungi have solved this problem by secreting hydrophobins into the aqueous environment. Assembly of hydrophobins at the interface between the moist substrate and the air results in the formation of an amphipathic film and, as a consequence, in a dramatic lowering of the water surface tension [19,22,23]. The process of formation of aerial structures has been well studied in *S. commune.* This basidiomycete forms a vegetative mycelium during the first three days of growth. During this period, the surface tension of the moist substrate is not changed and, as a consequence, the hyphae are forced to grow in the substrate only. At day four, the *SC3* gene is induced [24], possibly as the result of a signaling process. SC3 is secreted into the medium and will self-assemble at the interface between the medium and the air. This is accompanied by a decrease of the water surface tension from 72 to 24 mJ·m^−2^ [19]. A strain lacking *SC3* (*ΔSC3*) reduces the surface tension less dramatically and therefore forms only a few aerial hyphae [19,25]. Hyphae that grow into the air also express hydrophobin genes. The hydrophobins secreted by these hyphae cannot diffuse into the medium. Instead, they self-assemble at the interface between the hydrophilic cell wall and the air [26,27]. In this way, aerial hyphae [23,26,27], fruiting bodies [28], and spores [29,30,31,32] become hydrophobic. In case of aerial hyphae and fruiting bodies, surface hydrophobicity prevents these aerial structures to fall back into the moist substrate [26,27] and it may protect against bacterial and fungal infections [16]. Moreover, it prevents water to enter the gas channels in fruiting bodies [33]. In the case of spores, surface hydrophobicity facilitates dispersal of these reproductive structures by wind and insects [29,30,34] and it prevents desiccation [34]. Moreover, it plays a role in infection. The hydrophobin layer prevents immune recognition of conidiospores [35] and their clearance by neutrophils and macrophages in early stages of infection [36,37,38].

In addition to their role in aerial growth and reproduction, hydrophobins mediate fungal attachment to hydrophobic surfaces [39,40,41,42]. The hydrophobic conidiospores that are dispersed by wind or insects easily adhere to water-repellent biotic or abiotic substrates. Germlings resulting from these spores also secrete hydrophobins. These hydrophobins will self-assemble at the interface between the hydrophobic substrate and the cell wall. The *ΔSC3* strain of *S. commune* showed decreased attachment of hyphae to hydrophobic surfaces such as Teflon [39]. Similarly, a strain of the rice pathogen *Magnaporthe grisea,* in which the *mpg1* hydrophobin gene was inactivated, adhered less to the surface of its host. This reduced attachment affected formation of appressoria and infection [40,43,44]. Expression of hydrophobin genes during the infection process is probably widespread in pathogenic fungi. For instance, expression of hydrophobins has also been shown to occur in the tomato pathogen *Cladosporium fulvum* [45]. Apart from pathogenic interactions, hydrophobin-mediated attachment seems also to be essential in symbiotic interactions such as in lichens and mycorrhizas [16].

Hydrophobins also play a role in the architecture of the hyphal cell wall by influencing the linkage of glucan to chitin [23,46]. This effect was best studied in *S. commune.* Juvenile *S. commune* cultures, not yet expressing *SC3*, contain a cell wall composition similar to the *ΔSC3* strain. This wall contains a high amount of water-soluble glucan, whereas cell wall glucan of cultures expressing *SC3* becomes insoluble due to linkage to chitin [46].

In addition to the different biological roles fulfilled by hydrophobins, differences in temporal and/or spatial expression between members of hydrophobin gene families are observed, suggesting the possibility of functional specialization [47].

## 3. Interfacial Self-Assembly of Hydrophobins

Hydrophobins are capable of self-assembly into an amphiphilic film at hydrophilic-hydrophobic interfaces [12]. Examples are interfaces between water and air, water and oil and water and hydrophobic solids like Teflon. As mentioned, based on the spacing of the cysteine residues and their biophysical properties, hydrophobins can be divided in two classes [21]. Class I hydrophobins assemble into a protein membrane that can only be dissociated using trifluoroacetic acid and formic acid [26,48]. In contrast, assemblages of class II hydrophobins can be dissociated in 60% ethanol, 2% SDS [12,49,50] or simply by applying pressure [49]. By self-assembly, hydrophobins can change the surface of a hydrophilic material into a highly hydrophobic one, whereas hydrophobic material can be made moderately to highly hydrophilic. Coatings on hydrophilic surfaces can be obtained by drying down a hydrophobin solution [12]. The degree of hydrophobicity of the resulting coating is similar within class I hydrophobins (water contact angle ±120 degrees; Table 1). The hydrophobic side of class II hydrophobins seems to be less water repellent with a water contact angle ranging between 60 and 105 degrees. It can, however, not be excluded that these values are an under-estimation because of the lower stability of the class II hydrophobin membranes. Coatings on hydrophobic surfaces can be obtained by submerging or suspending the material into an aqueous hydrophobin solution. The wettability of the coating depends on the hydrophobin used. In the case of natural class I hydrophobins it ranges between 36 and 63 degrees, while in the case of the class II hydrophobins water contact angles are between 22 and 60 degrees (Table 1).

## 4. Structure of Class I and II Hydrophobins

Hydrophobins are about 70–120 amino acids in length. Their sequences are not highly conserved, not even within class I or II. Despite this, the structure of the hydrophobins seems to be the same [51,52,53,54]. Hydrophobins contain eight conserved cysteine residues which form four disulphide bridges [52,55,56]. The cysteine residues in SC3 are important to keep the protein in the soluble state [57]. In fact, reduction of the cysteine residues resulted in spontaneous or premature self-assembly in water. As a result, insoluble aggregates were formed in the aqueous environment [57]. Replacement of the cysteine residues in the class I hydrophobin MPG1 of *M*. *grisea* by alanine residues resulted in decreased secretion of the hydrophobin [54]. This is probably due to premature self-assembly of MPG1 during the secretion process. Thus, the cysteine residues seem to be important to confine the self-assembly process to hydrophilic-hydrophobic interfaces.

Hydrophobins can be modified post-translationally. For instance, the N-terminal part of secreted SC3 contains 16–22 mannose units. These O-linked sugar molecules influence the properties of the hydrophilic side of the assembled class I hydrophobin [58,59]. Deglycosylated SC3 does self-assemble on a hydrophilic-hydrophobic interface but the wettability at the hydrophilic side is decreased [59].

**Table 1:** Physiochemical properties of natural and engineered class I and class II hydrophobins. Surface activity measurements and coatings were performed at 100 µg·mL^−1^ unless mentioned otherwise. ND, not determined; ^a^22 µg·mL^−1^; ^b^80 µg·mL^−1^; ^c^coating not homogenous.

Hydrophobin	Fungus	Surface activity (mJ·m^-2^)	Hydrophilic side (θ)	Hydrophobic side (θ)	Rodlets	Reference
***Class I***						
SC3	*S. commune*	27–32	36 ± 3	115 ± 12	yes	[12,20,58]
deglycosylated SC3^a^	*S. commune*	32	66 ± 6	ND	ND	[58]
RGD-SC3	*S. commune*	32	44 ± 2	122 ± 4	yes	[58]
TrSC3	*S. commune*	32	73 ± 3	119 ± 3	yes	[58]
RGD-TrSC3	*S. commune*	30	68 ± 3	120 ± 3	yes	[58]
SC4	*S. commune*	35	48 ± 3	115 ± 3	yes	[36,98]
ABH1	*A. bisporus*	ND	63 ± 8	113 ± 4	yes	[31]
ABH3	*A. bisporus*	37	59 ± 5	117 ± 3	yes	[26]
HGFI^b^	*G. frondosa*	45	62 ± 2.5	ND	yes	[91]
***Class II***						
HFBI	*T. reesei*	42	59 ± 13	60–64	no	[20]
HFBII	*T. reesei*	35	-	60–70	no	[20]
CRP	*C. parasitica*	32	22 ± 2	≥90^c^	no	[12,25]
CFTH1	*C. fusiformis*	33	60 ± 5	105 ± 2	no	[99]

**Figure 1:**
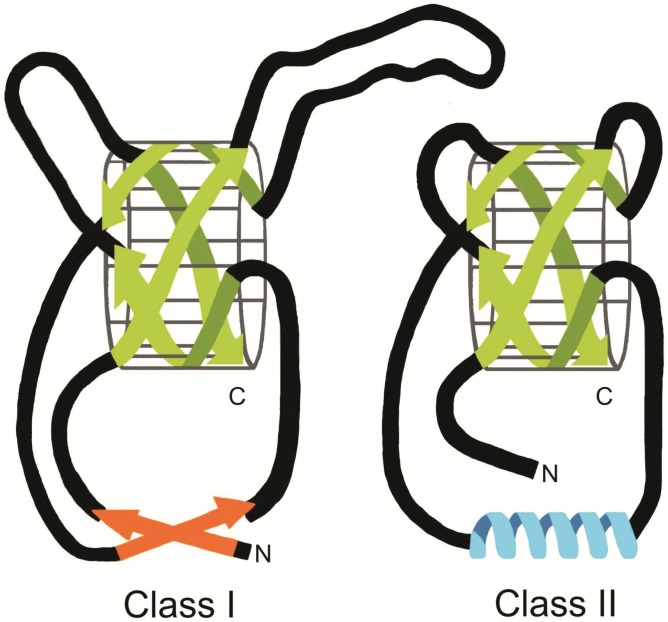
Schematic representation of the three-dimensional structure of class I and class II hydrophobins. Both types of hydrophobins contain a four-stranded β-barrel core. In class I hydrophobins two large disordered regions are present which are absent in class II hydrophobins. Finally, class I hydrophobins contain an additional two-stranded β-sheet structure, in class II hydrophobins this position is occupied by an α-helix.

### 4.1. Conformational Changes during Self-Assembly at Hydrophilic-Hydrophobic Interface

#### 4.1.1. Class I hydrophobins

The structure of the water soluble form of the class I hydrophobin EAS of *Neurospora crassa* has been solved [51]. It consists of a four-stranded β-barrel core, an additional two-stranded β-sheet and two sizeable disordered regions (Figure 1). EAS is cross-linked by the four disulphide bridges connecting C1–C6, C2–C5, C3–C4 and C7–C8. Notably, the charged residues are localized at one side of the surface of the protein. This strongly suggests that the water-soluble form of EAS is amphipathic. The largest disordered region of EAS (M22-S42) is contained between the third and the fourth cysteine residue. This part is the least conserved portion of class I hydrophobins in terms of both size and make-up. Importantly, the disordered regions of EAS do not seem to be important in the self-assembly process. Mutated EAS, in which half of the largest disordered region was deleted, was still able to self-assemble [51].

At a concentration of a few micrograms per milliliter or less, SC3 is in its monomeric form. At higher concentrations (starting at about 4 μg·mL^−1^), SC3 is mainly in a dimeric form [60,61]. Water-soluble SC3 contains about 23% α-helical state, 40% β-sheet structure, and 16% β-turn [58]. Self-assembly proceeds through two intermediate forms, *i.e*., the α-helical state and the β-sheet 1 state, to the stable β-sheet 2 state end form [62,63]. The α-helical content of SC3 increases during formation of the α-helical state, while random coil structures decrease [62]. Upon transfer to the β-sheet 1 state, the content of β-sheet structures increases to 65%. This is accompanied by the formation of a mechanically stable protein film, which has no clear ultrastructure. Changes in the secondary structure have not been observed during the transition to the β-sheet 2 state. However, during this transition SC3 forms 10 nm wide fibrils, which are known as rodlets. The rodlets of SC3 consist of two tracks, each made up of two to three 2.5 nm wide protofilaments [12]. Ellipsometry measurements have shown that the SC3 film is about 3 nm thick [63]. This and the fact that the diameter of the β-barrel of EAS is approximately 2.5 nm suggest that the rodlets are a molecular monolayer [51]. The charged patch on the surface of EAS would face the hydrophilic side of the interface, while the hydrophobic diametrically opposite site would face the hydrophobic side of the interface. This arrangement is consistent with the way other surface active molecules orient themselves at hydrophilic-hydrophobic interfaces [51]. The rodlets of SC3 and other class I hydrophobins are amyloid-like. They bind Congo-Red and Thioflavin T, and show the typical X-ray diffraction pattern of amyloids [12,51,64,65]. The amyloid-like fibrils of SC3 form a semi-permeable protein film with a cut-off of 200 Da [63]. In nature, this would allow translocation of amino acids, a few fatty acids and monosaccharides, but not of oligomers of these compounds or nucleic acids.

**Figure 2:**
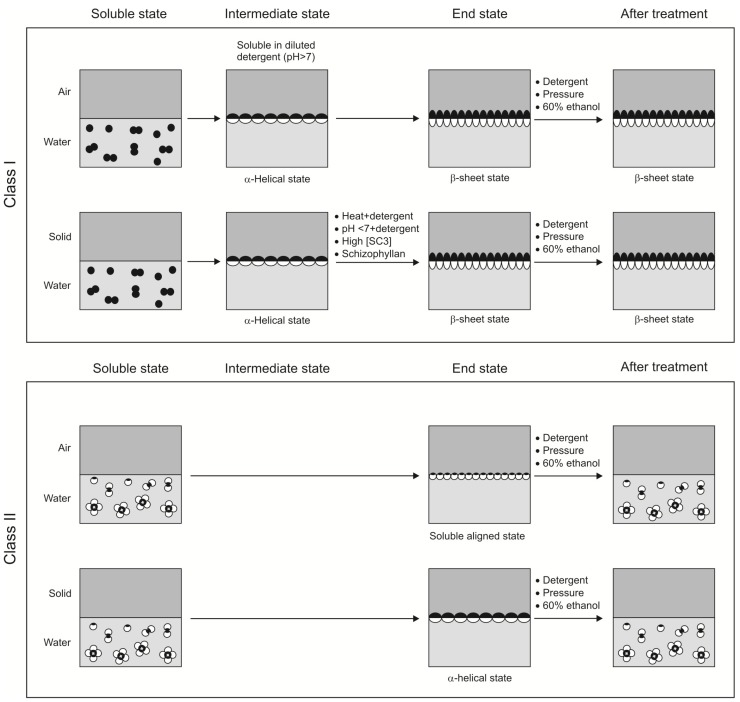
Model for assembly of class I and II hydrophobins at a hydrophilic-hydrophobic interface. At a water-air interface, class I hydrophobins (e.g., SC3; upper panel) spontaneously self-assemble via an α-helical intermediate state into a stable β-sheet end configuration. In contrast, upon contact with hydrophobic solids (e.g., Teflon) in water, SC3 is arrested in the intermediate α-helical configuration. The transition to the stable β-sheet end form is promoted by high protein concentration, presence of the polysaccharide schizophyllan (SPG) and the combination of heat or low pH and detergents. Class II hydrophobins (lower panel) do not assemble via an intermediate form. At the water-air interface, the conformation remains the same compared to the soluble state. The molecules orient themselves at the interface with the hydrophobic patch directed towards the air and the hydrophilic part directed to the water (soluble aligned state). On a solid-water interface, a conformational change into an α-helical form is observed. The end state of class I hydrophobins (upper panel) is very stable and cannot be dissociated by pressure, detergent or 60% ethanol. In contrast, the end form of class II hydrophobins (lower panel) readily dissolves under these conditions.

During self-assembly at the water-air interface, the structure of SC3 proceeds through the α-helical state to the β-sheet 1 state within a few minutes (Figure 2). Conversion to the β-sheet 2 state, however, takes several hours. Notably, self assembly of SC3 is arrested in the α-helical state on a Teflon surface when concentrations of ≤100 μg·mL^−1^ are used [58]. This form can be easily removed from the surface using diluted detergent at neutral pH (Figure 2). However, the combination of diluted detergent and high temperature or low pH [62,66] induces the α-helical form to proceed to the β-sheet 2 state (Figure 2). Recently, it was shown that diluted detergent and high temperature or low pH are not the only conditions that promote formation of the β-sheet 2 state at a hydrophobic solid. This state can also be attained by high SC3 concentration (300 μg·mL^−1^) and a long incubation time of 16 h. The β-sheet 2 state is also promoted by the presence of the cell wall polysaccharide schizophyllan (SPG) [66]. In this case, a concentration of 1 μg·mL^−1^ was sufficient to have SC3 adopt its stable end form. SC3 in the β-sheet 2 state cannot be removed from a hydrophobic solid with detergent at any temperature or pH [62,66]. The SC3 coating on a hydrophobic solid is therefore highly stable (Figure 2). The interaction of SC3 with a hydrophobic solid is less strong after deglycosylation of the protein [58]. This suggests that the mannose units are important for the strength of the interaction with the hydrophobic surface [58]. This effect is expected to be indirect since the mannose residues reside at the hydrophilic side of the molecule.

#### 4.1.2. Class II hydrophobins

The structures of monomeric HFBI and HFBII have been solved [52,53,54,56]. They have a near globular form of about 2 nm in diameter. Like the class I hydrophobin EAS, these proteins consist of a core with a β-barrel structure (Figure 1). However, HFBI and HFBII do not contain the two disordered loops found in EAS. Furthermore, the additional two-stranded β-sheet in EAS is replaced with an α-helix in the class II hydrophobins. This helix occupies basically the same region of space as the small sheet in EAS. The structure of HFBI and HFBII is cross-linked by the four disulfide bridges in the same way as in the class I hydrophobin EAS (C1–C6, C2–C5, C3–C4, C7–C8) [51,52,53,54]. One side of the monomer surface contains only aliphatic side chains. This creates a hydrophobic patch of about 4 nm^2^ to the otherwise fairly hydrophilic surface of the monomer. It has been proposed that this hydrophobic patch contributes to the enormous surface activity of class II hydrophobins [67].

Like SC3, HFBI and HFBII exist as monomers at a concentration of a few μg·mL^−1^ [67]. When the concentration is increased, HFBI and HFBII form dimers and at higher concentrations (*i.e.*, 0.5–10 mg mL^−1^) they form tetramers [67,68]. The monomers seem to have a higher affinity for surfaces than for formation of oligomers [15,67]. This supports the model [52] in which the hydrophobic patches of the monomers are shielded in solution by the formation of oligomers. These oligomers would dissociate at a hydrophilic-hydrophobic interface, which would result in the formation of a film which consists of a monolayer of the class II hydrophobin. However, a genetically engineered HFBI variant that forms native-like tetramers even at very low protein concentrations (*i.e.*, in the μg·mL^−1^ range) adsorbed to the air-water interface and lowered the surface tension of water in a similar way as HFBI [69]. This suggests that dissociation into monomers is not necessary for adsorption.

In contrast to SC3, self-assembly of HFBI and HFBII at the water-air interface is neither accompanied by a change in secondary structure nor by a change in ultrastructure [20]. This, and the fact that maximal lowering of the water surface tension was obtained within minutes [20], indicates that HFBI and HFBII assemble at the water-air interface as a monolayer with a structure similar to that in the water-soluble form (Figure 2). The hydrophobic patch at the surface of the class II hydrophobin monomers would explain such a behavior. Indeed, AFM studies also indicated a mono-molecular layer. This layer is not amyloid-like as in the case of class I hydrophobins and it also does not exhibit another clear ultrastructure [56]. Yet, the mono-molecular HFBI and HFBII layers were found to be highly crystalline. The experimental data suggest that four class II hydrophobin monomers form a tetramer, which further pack into crystalline domains. A torus-like shape was proposed for the HFBI tetramers, whereas a four-armed shape was proposed for the HFBII tetramers [70]. These different structures would explain the different properties of these hydrophobins. For instance, oil emulsions prepared with HFBI are more stable than those of HFBII, and HFBI interacts more strongly with Teflon making it wettable [20]. Interestingly, interaction of HFBI and HFBII with Teflon is accompanied with a change in the circular dichroism spectra, indicating the formation of an α-helical structure [20] (Figure 2). This change in structure has not yet been explained at a molecular level.

The class I hydrophobin SC3, did not affect self-assembly of the class II hydrophobins HFBI and HFBII and *vice versa*. When SC3 and HFBI or HFBII were mixed and dried down, islands of rodlets were observed surrounded by hydrophobin without an apparent ultrastructure [20]. It was concluded that the class II hydrophobins do not abolish, or at least not completely, self-assembly of SC3. It was argued that they compete for the available interface. Indeed, when mixtures of SC3 and HFBI were exposed to Teflon, water contact angles were obtained intermediate to those of pure SC3 and HFBI [20]. Yet, the class I and class II hydrophobins do somehow interact since precipitation of assembled SC3 by centrifugation was reduced by the class II hydrophobins.

### 4.2. Engineered Hydrophobins

As mentioned above, hydrophobin films exhibit a water contact angle at the hydrophilic side ranging between 22 and 65 degrees, whereas the hydrophobic side is typified by a water contact angle of 60–122 degrees [12] (Table 1). Thus, depending on the optimal surface wettability for a certain application (see below), one can choose for a certain class I or class II hydrophobin. The optimal biophysical and biochemical properties of hydrophobin films can also be obtained by genetic engineering. Engineering the N-terminal part of SC3 results in a change of the biophysical properties of the hydrophilic side of the assembled hydrophobin [58,59]. Deleting 25 of the 31 N-terminal amino acids preceding the first cysteine residue of SC3 (Gly29-Gly53) resulted in a truncated SC3 derivative, TrSC3, which lacks mannose residues. TrSC3 still assembles at hydrophilic-hydrophobic interfaces into an amphipathic membrane consisting of a mosaic of paired rodlets. These rodlets have a diameter of 8 nm instead of 10 nm for SC3. The hydrophobicity of TrSC3 at the hydrophobic side was similar to SC3. In contrast, the hydrophilic side was less wettable showing an increase of the water contact angle from 40 to 73 degrees (Table 1). Amino acids were also added to the N-terminal region of mature SC3 and TrSC3. Inserting the human fibronectin cell-binding domain (RGD) resulted in the hydrophobins RGD-SC3 and RGD-TrSC3. The biophysical properties of these hydrophobins were similar to that of SC3 and TrSC3 (Table 1).

The HFBI hydrophobin has also been fused to peptides and even proteins. GFP was functionally produced when fused to the N-terminal or C-terminal side of HFBI [71,72]. In the latter case, a flag epitope tag was placed at the N-terminus of HFBI. Similarly, HFBI was fused to the N-terminal part of a cellulose binding domain [73] and to the C-terminal parts of endoglucanase I, avidin and glucose oxidase (GOx) [71,72,73]. The catalytic activity of the GOx-HFBI fusion was shown to be similar to the commercial *Aspergillus niger* GOx reference [71]. Moreover, in all cases the fusion proteins, like native HFBI, could be purified by using aqueous two-phase system (ATPS) (see below). This shows that the amphiphilic nature of HFBI is not affected by a C-terminal or N-terminal fusion of the protein. Furthermore, a conjugate of cationic dendrons and an engineered HFBI (NCysHFBI; containing an additional Cys residue at the N-terminus) combines the adhesion properties of the class II hydrophobin with the dendrons DNA binding property [74]. The conjugate shows a high efficiency in DNA transfection experiments [75]. Finally, gold nanoparticles selectively interacted with a surface on which NCysHFBI was assembled [76].

## 5. Applications

Hydrophobins can be used in applications involving liquids and solid surfaces [12,13,14,16,47,77,78]. They can be used to improve the biophysical properties of a surface or can be used as a tag for other proteins. In this way, proteins can be immobilized on a surface or purified from a liquid.

### 5.1. Liquids

Class II hydrophobins, such as HFBI, show high separation behavior in aqueous two-phase systems (ATPS). Such liquid-liquid extractions can be used to purify proteins at large scale, especially when thermo-separating polymers and surfactants are used. Partitioning of a protein in one of the phases is not well understood but is assumed to depend on surface charge and hydrophobicity. The purification efficiency, as in other methods, depends on the properties of the other proteins in the mixture. The class II hydrophobin HFBI was used as a C-terminal or N-terminal tag to purify the cellulase endoglucanase I (EGI) and the cellulose-binding domains from the cellobiohydrolases CBHI and CBHII using ATPS [73]. These proteins were purified from the culture medium of the filamentous fungus *T. reesei,* which contains typically tens of different enzymes, by mixing with a non-ionic surfactant. These surfactants, C11EO2 and C12-18EO5, separate from the liquid culture medium above a certain temperature (*i.e.*, 9 and 19 °C, respectively) without the need for centrifugation. The amphiphilic nature of HFBI made that the fusion proteins partitioned into the surfactant phase, which makes up only 10–20% of the total volume. As a result, the protein was both concentrated and purified from the other proteins in the medium. In the next step, the surfactant was removed using extraction with isobutyl alcohol, leaving an aqueous solution of purified fusion protein. The EGI and cellulose binding proteins could be split from the hydrophobin by using cyanogen bromide cleavage at an introduced methionine in the fusion protein [73]. A similar approach was followed to purify proteins from insect and plant extracts [71,72]. Thus, class II hydrophobins can be used to efficiently purify proteins from complex mixtures using ATPS.

So far, class I hydrophobins have not been used as a tag for ATPS purification. The property to form highly insoluble assemblages makes these proteins unsuitable for this kind of application. However, both class I and class II hydrophobins can be used to disperse hydrophobic solids (e.g., Teflon beads) or liquids (oils) in water [20,39,79]. Teflon particles are used in several industrial applications (e.g., coating, lubrificant, sealant), where they are dispersed in aqueous solutions. Usually, the dispersion in water is achieved by using non-ionic surfactants at high concentrations. However, the stability of the resulting Teflon dispersion is affected by the temperature and by the chemical composition of the environment. Class I hydrophobins, due to their stable assemblages at relatively low surface concentration, are ideal candidates as stabilizing agents for solids like Teflon [79]. Examples of the use of class I and class II hydrophobins to stabilize hydrophobic liquids in water are emulsions for cream and ointment products [14]. Furthermore, the self-assembly property of class I and class II hydrophobins has been used in formulation of water insoluble drugs for oral administration [80,81]. The bioavailability of the hydrophobic drugs cyclosporine A and nifedipine was increased two and six-fold, respectively, when SC3 was added to the drug suspension [80].

### 5.2. Solid Surfaces

Low-friction surfaces are required in various biomedical applications including catheters and guide-wires [82]. Low friction reduces injury to tissue and increases the time the device can be used. Low-friction surfaces for biomedical devices can be obtained with lubricants such as silicone oil, glycerin, or jelly-type materials. However, their weak adhesion to the biomaterial reduces their performance in time. Teflon also provides low friction but this fluoropolymer is known to be lowly biocompatible. As an alternative, polystyrene (PS) and a copolymer of benzoyl-1,4 phenylene and 1,3-phenylene (PBP) were coated with SC3 [82]. Stable 10–20 nm thick coatings of SC3 were obtained on the polymers after spin coating or after adsorption of SC3 from an aqueous solution. Nanotribological analysis using Lateral Force Microscopy (LFM) showed ultralow relative friction coefficients for hydrophobin-coated surfaces. A reduction in the friction coefficient of 70–80% was obtained when compared to bare PS, while a 50–60% reduction was obtained when compared to bare PBP (note that PBP has a lower friction coefficient than PS). The coatings showed stable friction reduction over a period of several weeks.

Hydrophobins have also been exploited to pattern molecules or side groups on surfaces. Assembly of a class I hydrophobin from *P. ostreatus* was used to mask material in the KOH wet etch process [83], which is the basis of the silicon micromachining techniques. It was shown that the hydrophobin coating protected the silicon surface during the etching process. In other words, hydrophobins can be used to create chemical (nano)patterns on surfaces. This is also illustrated by the fact that gold nanoparticles selectively interacted with domains on a surface on which a genetically modified HFBI, NCysHFBI, was assembled [76].

Hydrophobins can also be used to adsorb proteins to surfaces without loosing activity. It was shown that several types of proteins (glucose oxidase from *A. niger*; bovine serum albumin; chicken egg avidin and monoclonal IgG1) adsorb onto a hydrophobic solid that was coated with the class I hydrophobin HGFI or the class II hydrophobin HFBI [84,85]. Efficiency of adsorption of these proteins on the hydrophobin layers depended on pH and ionic strength. Apparently, surface adhesion is due to selective charge interactions. Thus, hydrophobins can transform a non-polar surface into a polar one, and by this they can recruit proteins by charge interactions [85]. This principal has been used to immobilize enzymes in the development of biosensors [86,87,88,89,90]. The class I hydrophobin SC3 was used successfully in immobilization of glucose oxidase (GOx) and horseradish peroxidase (HRP) on glassy carbon electrodes [86]. The affinity of these enzymes for their substrate was similar when immobilized and dissolved enzymes were compared. Moreover, GOx was shown to maintain its activity for at least 90 days, even when the biosensor was used repeatedly. Similarly, HRP was still active on the 36th day after immobilization [86]. In principle, both class I and class II hydrophobins can be used to immobilize proteins. However, class I hydrophobins are preferred when detergents or pressure is used in the application.

Like proteins, cells can be immobilized on solid surfaces with the use of hydrophobins. Artificial materials can be used to replace or support a variety of body parts including bone, spinal, cardiac and dental tissues. The non-physiological character of these materials often leads to poor integration into human tissue and makes it necessary to develop implant materials that have improved biocompatibility. Hydrophobins can be used to improve the biocompatibility of implant materials. A hydrophobin optimally suited to coat a particular implant can be identified by screening the large variety of naturally occurring hydrophobins. Alternatively, hydrophobins can be modified by chemical cross-linking or genetic engineering [91]. In any case, a hydrophobin should not be immunogenic or toxic for use in a medical application. Low antibody titers, if any, were obtained when class I hydrophobins (e.g., SC3 and SC4 of *S. commune*) were injected subcutaneous into rabbits, indicating that hydrophobins are hardly immunogenic [91]. In fact, it has been suggested [16] and later shown [35] that, by covering fungal aerial structures, hydrophobins shield antigens in the cell wall, thereby protecting the fungal structure from the immune system. These observations indicate that the use of hydrophobins in medical applications will probably not elicit immunogenic reactions.

Growth of fibroblasts on Teflon served as the first model system to assess biocompatibility of hydrophobins [59,91,92]. Mouse fibroblasts grown on bare Teflon are round and not spread flat, indicating poor attachment. Coating with RGD-SC3, but not SC3, improves growth of the fibroblasts but TrSC3 was shown to be even better. TrSC3 not only increased cell numbers on Teflon, also the morphology of the cells was identical to that of cells grown on Tissue Culture Polystyrene. Similar to TrSC3, the natural hydrophobin SC4 promoted cell growth. These two hydrophobins share only 45% amino-acid identity but they form a less wettable coating compared to SC3. This suggests that the wettability is the determinant for promoting cell growth. Although cell growth was promoted, mitochondrial activities were affected by a coating with SC3 or SC4 in the α-helical state [91]. Interestingly, reduction of mitochondrial activity was negligible when SC3 and SC4 were in the β-sheet conformation [92]. As long as the significance of a reduced cellular activity is not clear, class I hydrophobins in the β-sheet conformation seem to be the preferred coatings. Class II hydrophobins have also been used to stimulate cell growth on solid surfaces [93]. A HFBI coating was used to adhere collagen to the hydrophobic surface of PDMS. The HFBI/collagen layer promoted adhesion and growth of human embryonic kidney cells. Similarly, growth of neural stem cells was promoted on micro-domains that had been coated with a HFBI/serum protein layer [94]. In this way, micro-patterns of neural stem cells were obtained on poly(lactic-co-glycolic acid) (PLGA) films. In other words, this method enabled controlled neural stem cell adhesion on the PLGA film.

## 6. Conclusions

By self-assembly, hydrophobins change the nature of a surface. This is of great importance in the life style of fungi, but can also be exploited in technical and medical applications. Hydrophobins can be used to change the wettability and/or the friction of a surface. Moreover, these amphipathic protein assemblages can be used to pattern surfaces with chemical groups or molecules. They can also be used to provide surfaces with a biocompatible layer that prevents denaturation of proteins and that promote cell growth. Industrial application of hydrophobins requires large scale production of these proteins. Gram per liter production of class II hydrophobins was achieved in *T. reesei* [95], but maximal production of class I hydrophobins in *S. commune* [19,77] and *Pichia pastoris* [96] was at least 10-fold less. Interestingly, a fusion of the class I hydrophobin DewA and (a truncated form of) yaaD of *Bacillus subtilis* was recently produced in *Escherichia coli.* Using a pilot plant, this resulted in kilogram scale purified hydrophobin [97]. These production levels will promote hydrophobins from proteins with potential, into proteins with applications. For these applications, one can choose from a palette of naturally occurring and engineered hydrophobins.
